# Vaccine sentiment analysis using BERT + NBSVM and geo-spatial approaches

**DOI:** 10.1007/s11227-023-05319-8

**Published:** 2023-05-07

**Authors:** Areeba Umair, Elio Masciari, Muhammad Habib Ullah

**Affiliations:** Department of Electrical Engineering and Information Technology, University of Naples Federico II, Via Claudio 21, 80125 Naples, Campania Italy

**Keywords:** COVID vaccines, Sentiment analysis, Artificial intelligence, BERT, BERT + NBSVM, Spatial analysis, Buffering, NBSVM, Vaccine hesitancy

## Abstract

Since the spread of the coronavirus flu in 2019 (hereafter referred to as COVID-19), millions of people worldwide have been affected by the pandemic, which has significantly impacted our habits in various ways. In order to eradicate the disease, a great help came from unprecedentedly fast vaccines development along with strict preventive measures adoption like lockdown. Thus, world wide provisioning of vaccines was crucial in order to achieve the maximum immunization of population. However, the fast development of vaccines, driven by the urge of limiting the pandemic caused skeptical reactions by a vast amount of population. More specifically, the people’s hesitancy in getting vaccinated was an additional obstacle in fighting COVID-19. To ameliorate this scenario, it is important to understand people’s sentiments about vaccines in order to take proper actions to better inform the population. As a matter of fact, people continuously update their feelings and sentiments on social media, thus a proper analysis of those opinions is an important challenge for providing proper information to avoid misinformation. More in detail, sentiment analysis (Wankhade et al. in Artif Intell Rev 55(7):5731–5780, 2022. 10.1007/s10462-022-10144-1) is a powerful technique in natural language processing that enables the identification and classification of people feelings (mainly) in text data. It involves the use of machine learning algorithms and other computational techniques to analyze large volumes of text and determine whether they express positive, negative or neutral sentiment. Sentiment analysis is widely used in industries such as marketing, customer service, and healthcare, among others, to gain actionable insights from customer feedback, social media posts, and other forms of unstructured textual data. In this paper, Sentiment Analysis will be used to elaborate on people reaction to COVID-19 vaccines in order to provide useful insights to improve the correct understanding of their correct usage and possible advantages. In this paper, a framework that leverages artificial intelligence (AI) methods is proposed for classifying tweets based on their polarity values. We analyzed Twitter data related to COVID-19 vaccines after the most appropriate pre-processing on them. More specifically, we identified the word-cloud of negative, positive, and neutral words using an artificial intelligence tool to determine the sentiment of tweets. After this pre-processing step, we performed classification using the BERT + NBSVM model to classify people’s sentiments about vaccines. The reason for choosing to combine bidirectional encoder representations from transformers (BERT) and Naive Bayes and support vector machine (NBSVM ) can be understood by considering the limitation of BERT-based approaches, which only leverage encoder layers, resulting in lower performance on short texts like the ones used in our analysis. Such a limitation can be ameliorated by using Naive Bayes and Support Vector Machine approaches that are able to achieve higher performance in short text sentiment analysis. Thus, we took advantage of both BERT features and NBSVM features to define a flexible framework for our sentiment analysis goal related to vaccine sentiment identification. Moreover, we enrich our results with spatial analysis of the data by using geo-coding, visualization, and spatial correlation analysis to suggest the most suitable vaccination centers to users based on the sentiment analysis outcomes. In principle, we do not need to implement a distributed architecture to run our experiments as the available public data are not massive. However, we discuss a high-performance architecture that will be used if the collected data scales up dramatically. We compared our approach with the state-of-art methods by comparing most widely used metrics like Accuracy, Precision, Recall and *F*-measure. The proposed BERT + NBSVM outperformed alternative models by achieving 73% accuracy, 71% precision, 88% recall and 73% *F*-measure for classification of positive sentiments while 73% accuracy, 71% precision, 74% recall and 73% *F*-measure for classification of negative sentiments respectively. These promising results will be properly discussed in next sections. The use of artificial intelligence methods and social media analysis can lead to a better understanding of people’s reactions and opinions about any trending topic. However, in the case of health-related topics like COVID-19 vaccines, proper sentiment identification could be crucial for implementing public health policies. More in detail, the availability of useful findings on user opinions about vaccines can help policymakers design proper strategies and implement ad-hoc vaccination protocols according to people’s feelings, in order to provide better public service. To this end, we leveraged geospatial information to support effective recommendations for vaccination centers.

## Introduction

Coronavirus flu spread since December 2019 from the Chinese city of Wuhan [1]. The quick diffusion of this dangerous flu caused an infectious disease sadly known as COVID-19 [2, 3]. In March 2020, it was declared as pandemic by WHO (World Health Organization) as it infected people all over the globe. During the last years, several variants of coronavirus was discovered in India, UK, Greece, Chad and South Africa [4]. Many safety measures were proposed by different organizations, in order to reduce the spread of COVID-19, like wearing masks and maintaining social distance to cite a few. However, for long term prevention, the development of vaccines seems to be the most effective solution [5, 6]. A large body of pharmaceutical industries put their efforts for quick development of vaccines. However, the development of the vaccines was not the only challenge to be addressed. Indeed, the really fast availability of COVID-19 vaccines compared to usual timing for new drugs release on the market caused skeptical reactions among population. Indeed, many studies state that, unfortunately, the development of vaccines does not automatically guarantee that people will be eager to get vaccinated but rather show vaccine hesitancy [7]. More in details, vaccine hesitancy refers to the reluctance, doubts or concerns that some people may have about getting vaccinated, despite the availability of safe and effective vaccines. This can be due to a variety of reasons, including lack of trust in the safety and efficacy of vaccines, misinformation or rumors about vaccines, religious or cultural beliefs, past negative experiences with vaccines, and perceived risks and benefits of vaccination. Vaccine hesitancy can lead to lower vaccination rates within a population, which can in turn increase the risk of vaccine-preventable diseases outbreaks. It is important to address vaccine hesitancy with evidence-based information, public education campaigns and effective communication strategies to encourage uptake of vaccines and ensure that people are protected against diseases [8]. Thus, vaccine hesitancy has been an obstacle for limiting the COVID-19 spread in many countries.

As an example of such phenomenon, many surveys got to the result that people belonging to age group 45–60 are more oriented to not get themselves vaccinated [9]. Thus, in order to make the vaccines campaigns successful, it is crucial to analyse people’s feelings and opinion about them [10]. As a matter of fact, for a better understanding of vaccines related opinion in a population it is important to analyse the people’s sentiments [7]. Therefore, it is necessary to know about people’s reactions and sentiments in order to design proper vaccine policy and campaigns [11].

More in detail, people share their thoughts and opinions by commenting on news, expressing their opinions using tweets, or providing feedback using social media. Thus, this vast amount of data can be fruitfully leveraged for opinion mining and knowledge discovery using suitable processing of text and data [12, 13]. Among the most widely used social networks like Facebook, Instagram, Whats-app, WeChat to cite a few, Twitter is probably the most widely used to share feelings and thoughts worldwide [14, 15] due to the peculiar features of its short text messages called tweets. In this respect, tweets can be leveraged by policy makers to analyse people’s reactions about reality topics [16]. Thus, the analysis of social media data by AI methods plays a crucial role in sentiment analysis [12]. To perform effective sentiment analysis, finding the polarity of the text is a key task as the polarity states whether a given text, sentence or tweet is either positive, negative or neutral [17, 18].

Based on polarity identification, we performed sentiment analysis of people reactions to COVID-19 vaccination campaign. Our proposed model used Twitter data and apply suitable pre-processing steps. Based on tweet polarity we classify tweets using BERT+NBSVM model. The result of sentiment analysis can be leveraged to reduce vaccine hesitancy. In this respect, we performed a spatial analysis using various geo-spatial techniques, including buffering and the average nearest neighbor method in order to provide a proper vaccine center suggestion based on people reactions, e.g., People’s beliefs about a specific vaccine could influence their choice of one center over another.

Indeed, Geographic Information System (GIS) technologies have gained great popularity during the pandemic as they enhance user comprehension of disease spread on a large scale through effective visualization tools. GIS mapping facilities can be useful for investigating the relationship between disease diffusion and a specific region [13]. Hence, hidden information about disease spread can be unveiled by properly mapping its spatial aspects. In this paper, we leverage such visualization features to relate sentiment analysis with vaccine center locations to propose proper suggestions to users based on user opinions in a specific region.

Finally, as data regarding user sentiment could quickly scale up at high speed, we propose an architecture to achieve high performance in real-life scenarios. However, we would like to point out that comparing the performance of our high-performance architecture against other proposals is beyond the scope of this paper, which focuses on the modeling performance of our framework supported by a suitable computing architecture.

More in details, our goals are summarized below:

- we want to find polarity of tweets and classify them in seven categories i.e. strongly negative, mild negative, weak negative, neutral, strongly positive, mild positive, weakly negative;
- we design the word-clouds of tweets to perform effective classification of positive and negative sentiment using BERT+NBSVM model;
- We use geo-spatial approaches to provide information on vaccine centers, taking advantage of sentiment analysis aiming to reduce vaccine hesitancy.

The rest of the paper is organized as follows. Section 2 describes the state-of-the-art in the field of sentiment classification during COVID-19. Sections 3 and 4 explain the methodology and high performance framework we proposed for the classification of people’s sentiments using Twitter data. Results of the experiments and the discussion on the results are given in Sect. 5. Section 6 discusses the geo-spatial analysis performed on the vaccine dataset. Finally in Sect. 7 we draw our conclusion and highlight some promising future directions.

## Related work

Sentiment analysis is defined as the extraction of people’s opinions from text using Natural Language Processing (NLP) techniques [19]. In recent literature, researchers are highly motivated to extract the sentiments of people from text, mainly generated by social networks. There are various techniques for this purpose, such as the dictionary-based methodology and corpus-based methodology [20], using clustering methods [21], and correlation analysis [3].

Recently, we faced COVID-19 pandemic that heavily influenced people’s lifestyle. Due to this dramatic circumstances, many researchers started analysing people’s feeling about COVID-19 from different dimensions [22]. A quite intriguing research direction leverages aims at performing sentiment analysis on user reactions based on AI tools. In [23], researchers proposed Context-Guided BERT for Targeted Aspect-Based Sentiment Analysis. Their proposed approach involves two methods to integrate context into the BERT architecture: (1) a Context-Guided BERT (CGBERT) model that is adapted from their recent context-aware self-attention network, which they applied to Targeted Aspect-based sentiment analysis (TABSA); and (2) a novel Quasi-Attention Context-Guided BERT (QACG-BERT) model that learns quasi-attention weights in a compositional manner, enabling subtractive attention that is lacking in softmax-attention. A Sentiment Analysis Case Study on Twitter is proposed in [24]. The authors examined the expression of anti-Asian sentiment in tweets related to COVID-19. To achieve this, they fine-tuned a transformer language model to classify tweets as hateful, counter-hateful, or neutral. After filtering out irrelevant tweets, they applied their model and tracked the frequency of hateful tweets over time and their correlation with relevant news reports.

In [25], the authors proposed aspect-based sentiment analysis using opinion tree generation. The Opinion Tree Generation, aims to identify all sentiment elements within a review sentence and represent them in a semantic tree structure. The opinion tree provides a detailed representation of the sentence structure, including aspect terms, opinion words, and semantic relationships. This approach has the potential to reveal a more comprehensive aspect-level semantic structure, resulting in improved sentiment element extraction. In [26], modified BERT for target-oriented multimodal sentiment classification has been proposed. The proposed model, named TomBERT, is built upon the BERT architecture, which is widely used for obtaining contextualized word representations through its pre-trained model parameters from a large corpus. To further improve their model, they designed a target attention mechanism inspired by the self-attention mechanism, which automatically learns the alignment between opinion targets and images. This mechanism allowed them to assign appropriate attention weights to different regions in the associated images, resulting in target-sensitive visual representations. They, then stacked a set of self-attention layers on top of the intra-modality alignments to capture their inter-modality interactions. By using this approach, the model is able to achieve better alignment between textual and visual information.

In [27], the authors explore the potential of generative pre-trained transformer language models in identifying COVID-19 symptoms in Twitter posts through the use of few-shot learning. In [28], a system for analyzing public sentiments and discussions about COVID-19 via Twitter activities is proposed. The goal is to showcase the capabilities of TweetCOVID, a system that leverages publicly available tweets to analyze the impact of COVID-19 on the population. The system offers a variety of functionalities, such as data collection and processing, sentiment and emotion analysis, topic modeling, controversy tracking, and visualization. In addition, researchers presented three use cases that demonstrate the system’s potential applications, including analyzing sentiments and emotions, detecting COVID-19-related topics, and understanding the use of controversial terms.

In [29], authors proposed the use of ensemble learning and machine learning techniques for identifying hate speech during the COVID-19 pandemic. Twitter data was collected by using trending hashtags and Twitter Application Programming Interface (API). Tweets were manually annotated into two distinct categories based on various factors. Features such as Tweet Length, Bag of Words, and TF/IDF were extracted. The findings of the study indicate that the Decision Tree classifier demonstrated effectiveness in detecting hate speech. Compared to other commonly used machine learning classifiers, it achieved better performances. In [30], manual data annotation has been performed to select most pertinent features. In [31], data obtained using Twitter API have been analyzed by opinion lexicon-based approach and emotion lexicon-based approach. The former categorized the tweets’ sentiment into three categories, while the latter further refine them into eight categories.

In [32], the authors propose a new big data architecture that uses image and text data to improve the sentiment analysis task. The architecture is designed based on the three big data principles - Volume, Velocity, and Variety. They show the application of the proposed architecture through a pipeline that integrates facial recognition and sentiment analysis. The pipeline uses open-source tools like Hadoop, Spark, and Machine Learning libraries for streaming conversion, image pre-processing, and data analytics. The paper also discusses the implementation components and visualizations used for the facial recognition use case.

## An hybrid approach for sentiment analysis

As discussed above, current approaches combine Machine Learning and manual annotations for natural language processing tasks working on text corpora. More in details, manual approaches leverage pre-defined rules for classifying texts with respect to sentiment categories [22], while the machine learning approaches use (un-)supervised algorithms for this task. In this paper, we propose a hybrid approach for sentiment analysis.

### Our framework in a nutshell

**Fig. 1:**
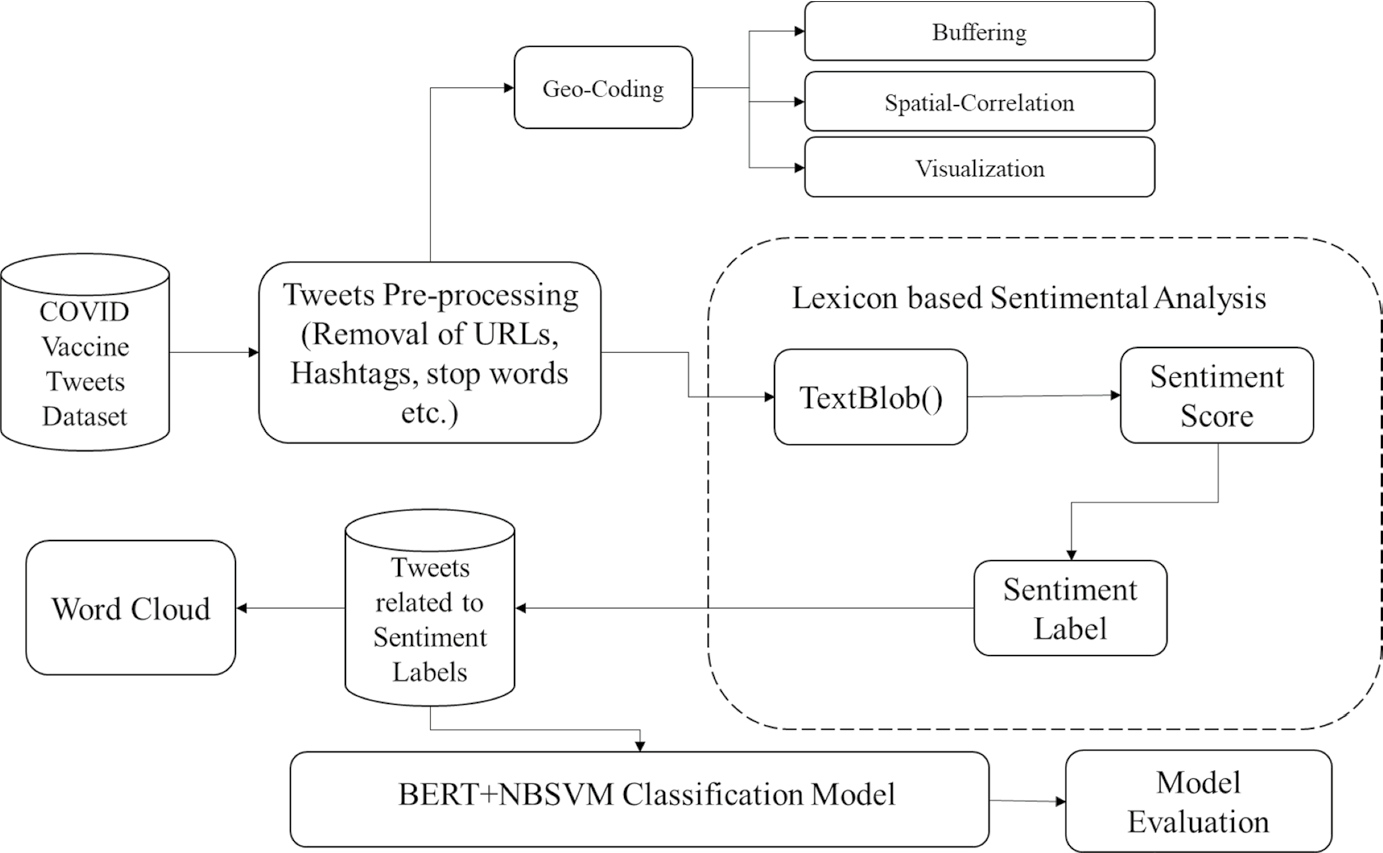
Our sentiment analysis framework

In order to properly address our sentiment analysis task related to COVID-19 tweets, we propose a methodology composed of the sub-tasks, as shown in Fig. 1.

First, we deal with dataset collection and pre-processing. Indeed, tweets are collected from their sources in the original unstructured format, thus we need to extract main features for subsequent analysis and eliminate unnecessary and noisy information like stop words and URLs to cite a few.

Sentiment classification is then performed by extracting the sentiments and their polarity value using lexicon based approaches. As polarity values are computed, we perform sentiment classification by our innovative BERT+NBSVM model. Finally, we apply geo-spatial methods in order to model the geographical distribution of the vaccine sentiments, which will be used to identify more appropriate vaccination centers based on the sentiments related to a specific region.

### Data collection

In our framework, we collected publicly available Twitter data from the Kaggle website. More specifically, the dataset is available on Kaggle website^1^ [33]. It collects recent tweets about COVID-19 vaccines distributed worldwide and is reported below for the sake of completeness:

- Pfizer/BioNTech;
- Sinopharm;
- Sinovac;
- Moderna;
- Oxford/AstraZeneca;
- Covaxin;
- Sputnik V.

We used Twitter API through the Tweepy Python package to collect tweets containing the terms Pfizer/BioNTech, Sinopharm, Sinovac, Moderna, Oxford/Astra-Zeneca, Covaxin, and Sputnik V. The attributes we extracted from the dataset and their description are reported below:

- ID: Unique identifier for each tweet
- user_name: Screen name or username of the Twitter account that posted the tweet
- user_location: Location listed on the user’s Twitter profile
- user_description: Bio or description listed on the user’s Twitter profile
- user_created: Date the user’s Twitter account was created
- user_followers: Number of followers for the user’s Twitter account
- user_friends: Number of accounts the user is following on Twitter
- user_favourites: Number of tweets the user has favorited on Twitter
- user_verified: Boolean indicating if the user’s Twitter account is verified
- date: Date and time the tweet was posted
- text: Content of the tweet
- hashtags: Any hashtags included in the tweet
- source: The device or application used to post the tweet
- retweets: Number of times the tweet has been retweeted
- favorites: Number of times the tweet has been favorited
- is_retweet: Boolean indicating if the tweet is a retweet or an original post.

### Data pre-processing

As it is easy to see we selected only relevant attributes for our analysis. To this end, the dataset has been cleaned by removing URLs from the text, removing the hashtag symbol from the tweets and finally removing stop-words. To perform this task we wrote ad-hoc Python scripts. In Table 1, we show some examples of the results obtained after tweets pre-processing.

**Table 1:** Comparison of tweets before and after pre-processing

Dummy samples	Hashtags removal	URLs removal
Fever after first dose #PfizerBioNTech https://t.co/ ffiee77	Fever after first dose PfizerBioNTech https://t.co/xffiee77	Fever after first dose PfizerBioNTech
Vaccine scheduling available online https://t.co/jgeeityc	Vaccine scheduling available online https://t.co/jgeeityc	Vaccine scheduling available online
Second dose done?? https://t.co/ooehdugy	Second dose done https://t.co/ooehdugy	Second dose done

### Getting sentiment polarity values

In order to perform a valuable sentiment analysis, polarity values computation for tweets is crucial. Polarity indicates whether a given sentence falls in positive category or negative category based on some pre-defined classification. We leveraged the classification proposed in [22] and categorized our tweets into seven sentiment classes based on the values of their sentiment polarity. The sentiment classes are defined as: *neutral, weakly positive, mild positive, strongly positive, weakly negative, mild negative and strongly negative*.

**Fig. 2:**
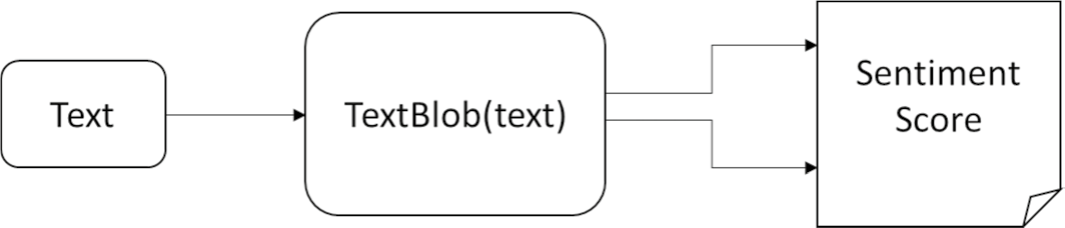
TextBlob() workflow

In order to compute the polarity values (that falls in the interval [− 1 to +1]) for each tweet we leveraged Python TextBlob() library function whose workflow is shown in Fig. 2.

The polarity values are then used to compute the overall classification of the tweet as positive, negative, or neutral. This information about the positive, negative, or neutral tweet classification can be used to generate word clouds. In detail, each word cloud assigns word sizes according to their occurrence frequencies. In a word cloud, a bigger word size denotes a higher number of occurrences of the word. We plotted the word cloud using Python scripts to observe the high-frequency words in positive, negative, and neutral clouds. This step is particularly useful since the most frequent words can help identify the dominant opinions and feelings of people regarding a specific vaccine issue.

### Combining BERT and Naive Bayes-SVM for sentiment classification

In this section, we propose a combination of BERT (Bidirectional Encoder Representations from Transformers) and NBSVM (a hybrid of Naive Bayes and Support Vector Machine) for sentiment classification of vaccine-related tweets. BERT is a well-established tool for transformers and attention mechanism implementation. The transformer is a sequence-to-sequence model based on attention mechanisms for encoding and decoding textual information. However, the BERT architecture does not properly utilize the decoder potential as it only leverages the encoder layer of the transformer [34, 35]. BERT has two main architectures: BERT Base and BERT Large, which exhibit some major differences in text modeling with respect to four main features, i.e., the number of hidden layers in the encoder, the number of self-attention heads, the hidden size of the feed-forward network, and the maximum sequence length parameter [34].

On the other hand, Naive Bayes is a machine learning algorithm that performs well for short text sentiment analysis, while SVM is more appropriate for longer text. We chose to implement a hybrid approach using both Naive Bayes and SVM to obtain higher accuracy by incorporating the log count ratio obtained by Naive Bayes as a feature value in SVM. This choice has been proven to be quite versatile for various analysis tasks and types of data collections [36].

### Leveraging BERT and NB-SVM synergies

**Fig. 3:**
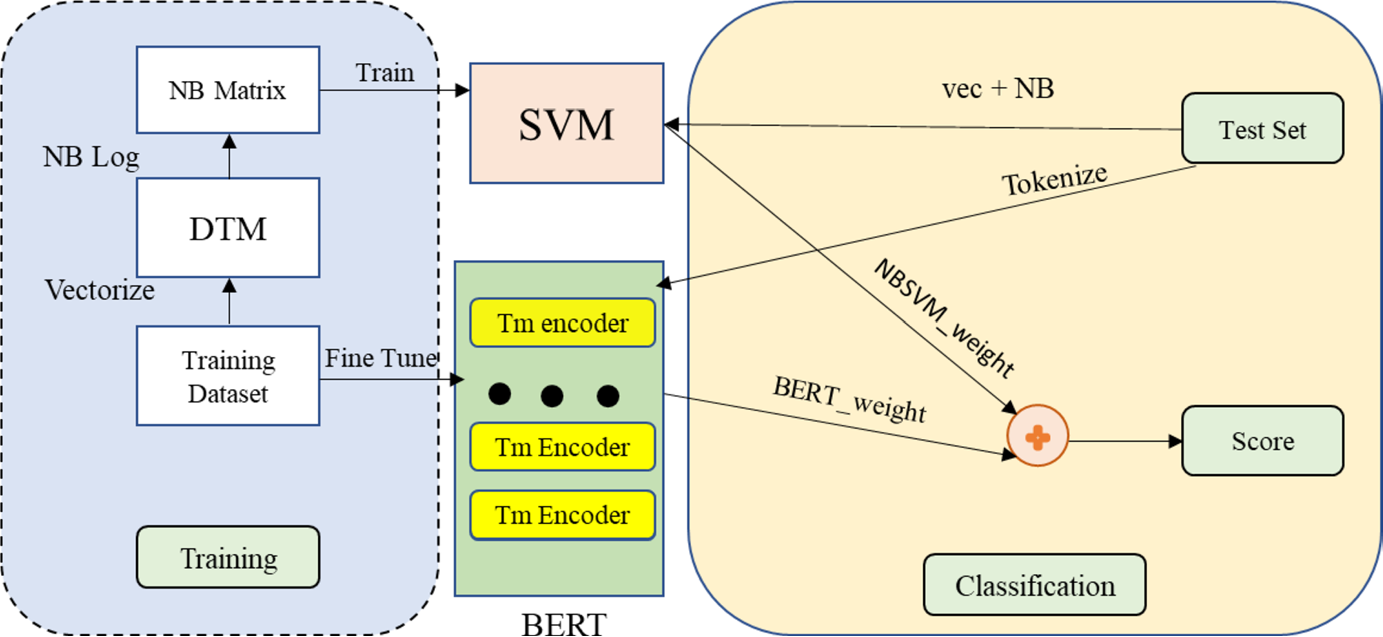
System architecture of BERT+ NBSVM

Our BERT+NB-SVM based architecture takes advantage of the regression fine-tuned sequence-pair obtained by BERT and leverages the Naive Bayes-Support Vector Machine (NB-SVM) model to obtain document-term matrices (DTM) that compute the Naive Bayes Log-count ratios. The latter model determines the probability that a given word appears in the document in positive versus negative classes. In a sense, we combine the strengths of deep learning and classical machine learning approaches to obtain a more accurate sentiment analysis. The system architecture of our BERT+NB-SVM based approach is depicted in Fig. 3.

Herein, we perform the following steps for training and classification:

1. We fine-tune the BERT model on the training dataset.
2. We train an SVM model using the log count ratios obtained by Naive Bayes.
3. The final score is computed as the weighted sum of the obtained NB-SVM model and the best fine-tuned BERT model (i.e., the BERT model that exhibits the best performance over different epochs and with different batch sizes).

### Hyper-parameters used for model training

We performed both pre-training and fine-tuning to develop our model. Specifically, we used the Adam optimizer as the loss function for training the model [37]. Then, we performed grid search for parameter tuning, and found the best weight for the BERT model to be 0.87, while for NB-SVM it was 0.08.

We trained one model for positive tweet classification, and a second one for the classification of negative tweets. To evaluate our model, we computed precision, recall, and $F_1$ score, which are defined in Eqs. 1, 2, and 3, respectively:

1 $$\begin{aligned} Precision = \frac{TP}{TP + FP} \end{aligned}$$

2 $$\begin{aligned} {\rm Recall} = \frac{{\rm TP}}{{\rm TP} + {\rm FN}} \end{aligned}$$

3 $$\begin{aligned} F\,{\rm Measure} = \frac{2 \times {\rm Precision} \times {\rm Recall}}{{\rm Precision} + {\rm Recall}} \end{aligned}$$

where (1) TP is true positive count and computes the number of positive prediction of positive instances, (2) TN is true negative count and computes the number of positive prediction of negative instances, (3) FP is false positive count and computes the number of negative prediction of positive instances and (4) FN is false negative count and computes the number of negative prediction of negative instances.

### State-of-the-art algorithms for experimental comparison

To evaluate and compare the results of our model, we designed an experimental comparison with the main state-of-the-art algorithms. We performed the comparison with KNN (*K*-nearest neighbor) algorithm, SVM (Support Vector Machine) algorithm, RF (Random Forest) algorithm, NB (Naive Bayes) algorithm, and DT (Decision Tree) algorithm because of their wide adoption [2, 12, 38].

Decision tree and random forest algorithms belong to the same family, and they are able to learn from user interests [39]. Random forest algorithms usually avoid some pre-defined assumptions and work well when the dataset is non-linear and contains high-order interactions. Random tree works by performing classification and regression trees and their votes. It chooses random samples as well as random features from the dataset and generates binary trees for training the model. It uses one-third of the class for testing [40].

On the other hand, decision tree generates simple decision rules from the entire dataset and trains its model on those rules to predict the class values. Decision tree algorithms are well-suited when the dataset is small [41]. Another widely used machine learning algorithm is Naive Bayes. It assigns equal weights to all features and considers them statistically independent, which means that the feature values do not exhibit any relationship [41, 42]. Naive Bayes calculates the probability of each feature using the Bayesian theorem reported in Eq. 4:

4 $$\begin{aligned} P (H|X) = P (X|H) P (H)/ P (X) \end{aligned}$$

where *H*: represents the sentiment class (positive or negative), *X*: represents the input data, such as a text document, *P*(*H*): represents the prior probability of the sentiment class, which is the probability of the sentiment class occurring without considering the input data, *P*(*X*): represents the prior probability of the input data, which is the probability of observing the input data without considering the sentiment class, *P*(*X*$\mid$*H*): represents the conditional probability of the input data given the sentiment class, which is the probability of observing the input data given that it belongs to a specific sentiment class, *P*(*H*$\mid$*X*): represents the posterior probability of the sentiment class given the input data, which is the probability of a particular sentiment class given the observed input data.

Another widely used algorithm is KNN that works by searching for the most similar instances in the whole dataset, which in turn requires a huge amount of time for processing. Hence, it should only be used with simpler and smaller datasets. The interesting fact about KNN is that it does not form a test-train model. Rather, it searches for the nearest value in the dataset. The parameter used for searching is the number of neighbors, which is provided by users [42]. KNN uses the formula reported in Eq. 5 to search for the similar sample:

5 $$\begin{aligned} di = \sqrt{[(xi-x)^2+(yi-y)^2]} \end{aligned}$$

where, di is the Euclidean distance between a point (*xi*, *yi*) and a reference point (*x*, *y*), *xi* is the *x*-coordinate of the point being considered, *yi* is the *y*-coordinate of the point being considered, *x* is the *x*-coordinate of the reference point, *y* is the *y*-coordinate of the reference point.

Support vector machine algorithms are quite effective in high-dimensional feature space. Indeed, SVM generates a hyperplane which is used for classification. SVM produces a single feature by combining features from different sources and then trains the model. The hyperplane with the largest separation between the points of two different classes is chosen for classification. SVM leverages linear, polynomial, sigmoid, and radial basis function (RBF) as kernel functions [43].

Finally, BERT (Bidirectional Encoder Representations from Transformers) works on masked language (MLM) by using a word representation model. BERT uses separation tokens [SEP] and classification tokens [CLS] and takes the [CLS] token as the initial input, which is further enriched by word sequences. It then transfers the input to upper layers, where the self-attention mechanism is applied. The result is then directed to the upcoming encoder through the feed-forward network. The obtained vector C represents the output of the model, which can be used for multiple purposes, such as classification and translation, to name a few. The probability of sentiment classes can then be computed by equation 6 [44]:

6 $$\begin{aligned} P = softmax(CW^T) \end{aligned}$$

where *P*: is the probability distribution over sentiment classes, where each element of P represents the probability of the input belonging to a particular sentiment class, softmax(): is a function that maps a vector of arbitrary real values to a probability distribution such that the output values are non-negative and sum up to 1, *C*: is a matrix that contains the learned representation of the input data, also known as the embedding matrix, *W*: is a matrix of learned weights that map the input representation to the sentiment class probabilities, *T*: is the transpose of the weight matrix *W*.

## Achieving high performances: the sigma architecture

In this section, a new architecture named *Sigma* is proposed to provide a solution for building a complete, interactive, and scalable Big Data System using a variety of tools and techniques that achieve high-performance execution of the framework defined so far for Sentiment and Geo-Spatial analysis. After testing our framework for limited-sized datasets, we tested it in a high-performance environment to make it suitable for real-life scenarios where data size quickly increases, thus requiring a proper architecture to deal with it. The typical working scenario collects data from real-time sources that need to be collected and analyzed properly, as they could quickly exceed common computational facilities, in order to make them suitable for the analysis steps. In order to address the aforementioned computational issues, we describe in this section, the Sigma Architecture that differs from well-known Lambda [45] and Kappa [46] architectures, which are considered the reference architectures for supporting the tasks described in our framework.

**Fig. 4:**
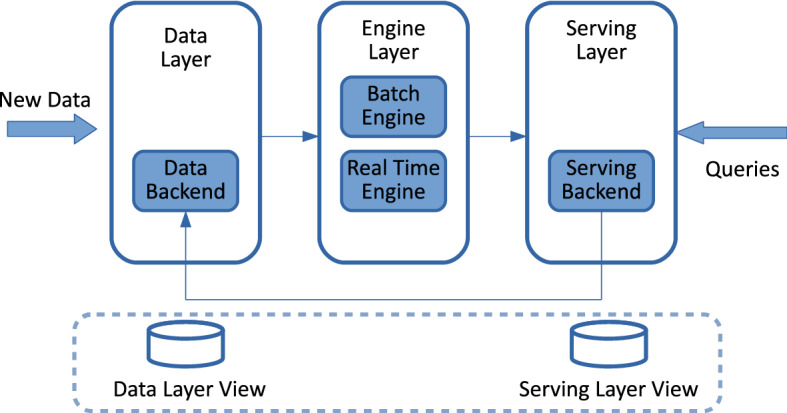
Sigma architecture

Sigma Architecture is composed of three Layer as depicted in Fig. 4.

The *Data Layer* stores a copy of all raw data that are collected by the system. This layer stores an immutable, constantly growing dataset (*Data Layer View*) and offer a back-end able to perform random reads on the whole content. The *Engine Layer* is responsible to compute arbitrary function on the data layer and to store results on the Serving Layer. This computation can be executed in batch mode on the whole Data Layer View (via Batch Engine) or can be executed in real time mode every time new data arrives in the Data Layer (via Real Time Engine). The *Serving Layer* is a specialized distributed database that loads in the results of Engine Layer Computation (*Serving Layer View*) and makes it possible to do random reads on it.

As regards new data coming into the system we may note that Big Data usually arise as streams, so the Data Layer need to serialize these flows and persist it. Moreover, in this step a first operation of Extract, Transform, and Load (ETL) is performed on incoming data flows. The Sigma Architecture allows to manage different situations. It is possible that data are already present in a data warehouse and in this case it is convenient to create the Serving Layer View using a batch operation via Batch Engine using for example the Map Reduce paradigm. In other situations, instead, data can only come in the form of streams and in this case it is convenient to create the Serving Layer View using real time functions via Real Time Engine. Maintaining all data on Data Layer allows to easily recover the system in case of failure on Serving Layer and allows to extends the Data Layer content by applying Batch Engine functions on the starting contents of the Data Layer. Once the Engine Layer created the Serving Layer View on the Serving Layer, the system is ready to receive queries via Serving Back-end. To satisfy incoming queries, the Serving Back-end uses data from Serving Layer View and data from Data Layer View, performing one or more requests to Data Back-end. This mechanism is useful to optimize the content of the Serving Layer allowing him to keep only an index or link for some data thus avoiding excessive data replication between Data Layer and Serving Layer. Moreover, with this mechanism, a list/detail view is straightforward simply to implements where: (1) *list* is the report or summary result of a query for the system that can be considered as a group of function on detailed data. This is the classical useful view for the knowledge of a system status in a given time interval; (2) *detail* contains all the information related to a single event (or group of event based on system granularity). This view is useful to analyze outlier or to quickly discover unwanted situations.

The Sigma Architecture can therefore be summarized by the following equations: 
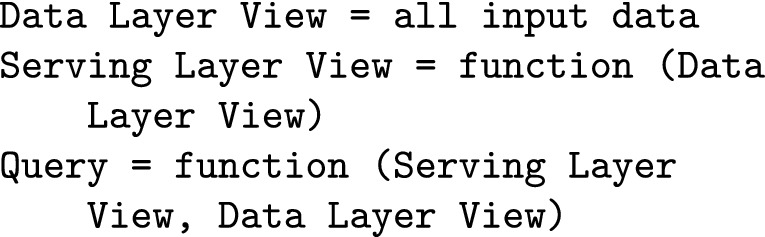


### Differences between lambda and sigma architectures

The Sigma Architecture differs from the Lambda Architecture in several aspects. Even though the Data Layer is similar to the Batch Layer, the Sigma Architecture provides data access from both the Engine Layer and the Serving Layer. For data analysis on the Serving Layer, the proposed architecture can operate either in Batch or Real Time Engine, but not both simultaneously as proposed in Lambda. Our choice has been made by considering that the Batch Engine is typically used when persistent data is available, there are system failures for system recovery, or there is a need to operate on the Data Layer. The Real-Time Engine, instead, is used for data flow elaboration. Based on this observation, an implementation of the Sigma Architecture could leverage either the Batch Engine or the Real-Time Engine. The decoupling between the Batch and Real-Time Engine allows us to considerably simplify the implementation and maintenance of the system, as this was one of the main criticisms of the Lambda Architecture.

### Differences between kappa and sigma architectures

As for the Kappa architecture, the proposed solution adds the Data Layer and enables batch processing. This layer allows us to decouple the input data from the Engine Layer, at the cost of slightly increasing the complexity of the system. However, it optimizes the data storage on the Serving Layer, reducing its size. Moreover, the use of Batch Engine allows for a higher number of instances of Sigma Architecture compared to Kappa. For example, when there is a need to analyze persistent data or perform batch operations on input data.

### A sigma basic implementation

To deeply understand the Sigma architecture two implementation will be showed. The first implementation is related to a scenario where data to analyze are persistent on a Big Data warehouse while in the second implementation data comes in form of flows.

#### Scenario 1. Persistent data

**Fig. 5:**
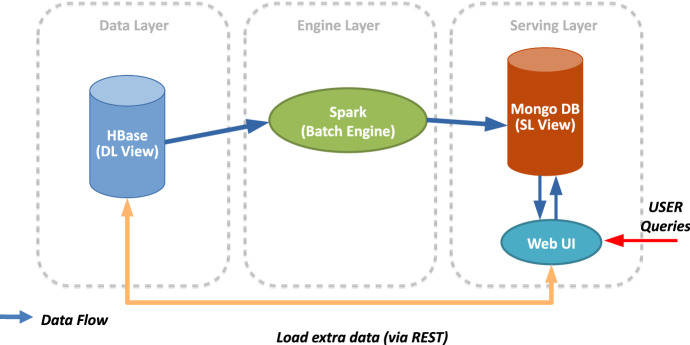
Sigma persistent data scenario

Figure 5 shows a possible implementation for a persistent data scenario. In this case, the whole dataset is fixed and stored in a Big Data Warehouse. The Data Layer is based on *Apache HBase*^2^ project. This tool is a column family based NoSQL datastorage modeled after Google’s Bigtable and written in Java. It allows to store a large amount of data by offering a scalable and fault-tolerant architecture, through addition of commodity nodes to cluster and native integration with MapReduce. It also provides access to resources through simple REST APIs and ability to work in memory to increase performance. This datastore offers the ability to perform random reads, which is a requirement for Sigma Data Layer. HBase uses a Column Family type data model, so applications that use it, store information in tables composed of rows and columns, whose intersections (cells) are versioned and can contain content of various kinds, formally considered an array of bytes. Row keys are also an array of bytes and can theoretically contain anything from strings to binary data. Rows in tables are ordered through row key that allows access to contained information through queries. Columns are grouped into column families and can be added at run-time (by specifying column family through a prefix). In addition to concept of column, table and row, HBase also uses the so-called regions. In fact, the tables in HBase are automatically partitioned horizontally into regions that are distributed in cluster. Each region includes a subset of rows from a table, in this way, a table that is too large to fit on one server can be distributed to different servers in cluster. In the parlance of Eric Brewer’s CAP Theorem, HBase is a CP type system.

The Engine Layer of this scenario is based on a *Apache Spark*^3^ that implements the Batch Engine Module of Sigma Architecture. Apache Spark is an open source distributed computing framework developed by the University of California’s Berkeley’s AMPLab and later donated to Apache Software Foundation. Characterized by the ability to store (usually partial) results in central memory, it offers a valid alternative to Map Reduce, which necessarily stores results of computations on disk. Apache Spark has as its architectural foundation the resilient distributed dataset (RDD), a read-only multi-set of data items distributed over a cluster of machines, that is maintained in a fault-tolerant way. Spark uses a master/slave architecture, where there is one coordinator process and many worker processes. The coordinator is named driver, while the worker is named executor. Since each execution takes place in a separate process, different applications cannot share data unless they are first written to disk.

With this framework it is possible to write any type of function to translate data from Data Layer View to Serving Layer View. In addition, Spark provides a well-assorted set of ready-made libraries for machine learning (MLib) and very large graph analysis (GraphX).

The Serving Layer is devoted to the interaction of end-users with the system and is based on the *MongoDB*^4^ project. MongoDB is a NoSQL, document-oriented database developed to obtain high reading and writing performance and the ability to be easily scalable through automatic failover. The document-oriented approach makes it possible to represent complex hierarchical relationships through the use of nested documents and arrays.

This database stores data in JSON format documents. JSON provides a data model that adapts perfectly to various types of programming languages and, since it has no fixed schema, it is much easier to expand the data model compared to relational databases that work on a fixed scheme. Moreover, it provides many properties of relational databases such as secondary indexes, dynamic queries, sorting, rich updates, upserts (updates that occur only if the document exists and are inserted if it does not exist), and aggregations.

Into the Serving Layer, Mongodb act as part of Serving Backend and as Serving Layer View container. We chose to use this datastorage system as the JSON document format allows on the one hand easier insertions for Engine Layer outputs and on the other hand fits perfectly with the "aggregate" structure of serving layer view.

Finally, the *Web UI* is a custom module that allows users to interact with the system. This modules perform random queries on MongoDB (for list visualization) and selection of record on HBase (for detailed visualization).

#### Scenario 2. Data flow

**Fig. 6:**
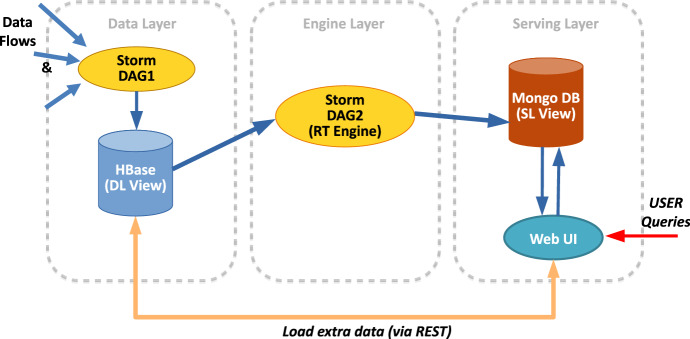
Sigma real-time data scenario

Figure 6 shows a possible implementation of the Sigma Architecture for a real-time data flow scenario. The structure is very similar to the scenario seen above, except for the Data Layer and the Engine Layer that utilize the *Apache Storm*^5^ Project. Storm is a distributed stream processing computation framework that processes raw data streams and provides several features such as fault tolerance, cluster balancing, etc. It uses the notion of “spouts” and “bolts” where a spout is a source of streams and a bolt consumes any number of input streams, performs some processing, and possibly emits new streams. A Storm application is designed as a directed acyclic graph (DAG) with spouts and bolts acting as the graph vertices. In the proposed scenario, we have 2 instances of Storm, one in the Data Layer and another one in the Engine Layer. It is just a conceptual separation, the same result can be achieved with just one instance that performs the following steps:

Takes the incoming input from the Data Layer as a data flow and stores it on the HBase data storage. Utilizes a number of "bolts" to process the incoming input and emits the results on the Mongo DB instance of the Serving Layer.

## Results and discussion

In this section, we will discuss the results we obtained by using the Sigma architecture in a Persistent Data setting as we collected the data in batches.^6^ Our setup provides a data collection service with the possibility of scaling out the computing cluster to manage the increase in the volume of data to be processed. The solution includes backup functionalities to manage the database saving even when the volumes managed grow suddenly. Overall, the computing cluster provides 8 TB of storage space, 8 vcores, and 120 GB of RAM. The execution service is guaranteed by 4 virtual machine instances having the following characteristics: 4 virtual cores with Xeon Broadwell processors, 30 GB of RAM, and 100 GB of SSD persistent storage.

### Sentiment polarity computing

The sentiment of a text can be determined by its polarity value. Table 2 displays the polarity values and their corresponding category types for each sentence, as defined in Sect. 3.

**Table 2:** Polarity values and sentiment categories with respect to sample tweets

Tweet sample	Polarity	Sentiment
Fever after first dose PfizerBioNTech	− 0.5	Mild negative
Vaccine scheduling available online	0.7	Strongly positive
Second dose done	0	Neutral

In order to validate our approach, the sentiment polarity was assessed by linguistic experts who validated our assignments through a questionnaire administered to a cohort of one hundred volunteers, uniformly distributed across an 18–70 age group.

### Word cloud computing

**Fig. 7:**
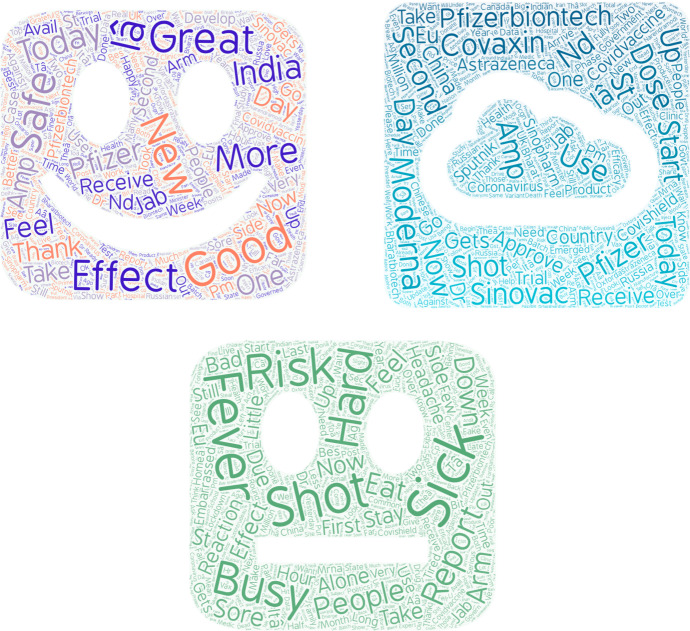
Figure contains Positive word cloud of tweets, Negative word cloud of tweets and the neutral word cloud of tweets

To accomplish our task, we computed the word clouds of the positive, negative, and neutral tweets. For this purpose, we first classified the data into three subsets using the polarity values of the tweets. Figure 7 displays three different word clouds for positive, neutral, and negative words. Some categories, along with their respective words, are given below:

- Words like “great”, “Good”, “More”, “Safe”, “Thank”, “Better”, “Happy”, “Love” may indicate that people are willing to get vaccinated.^7^
- Words like “Sick”, “Fever”, “Risk”, “Hard”, “Bad”, “Out”, “Serious” show that people are not happy with the vaccination campaign.
- Few words like “COVID”, “Shot”, “One”, “Second”, “People”, “Country”, “Pfizer/BioNtech”, “Astrazeneca”, “Sinovac”, “Sinopharm”, “Moderna” do not show any emotion related to vaccines.
- Words like “Receive”, “Batch”, “Dose”, “Russia”, “China”, “Vaccine”, “EU”, “Take”, are neither positive nor negative.
- Few words like “Alone”, “Long”, “Fail”, “Still” show that people are feeling anxious during vaccination.

### Sentiment classification

**Fig. 8:**
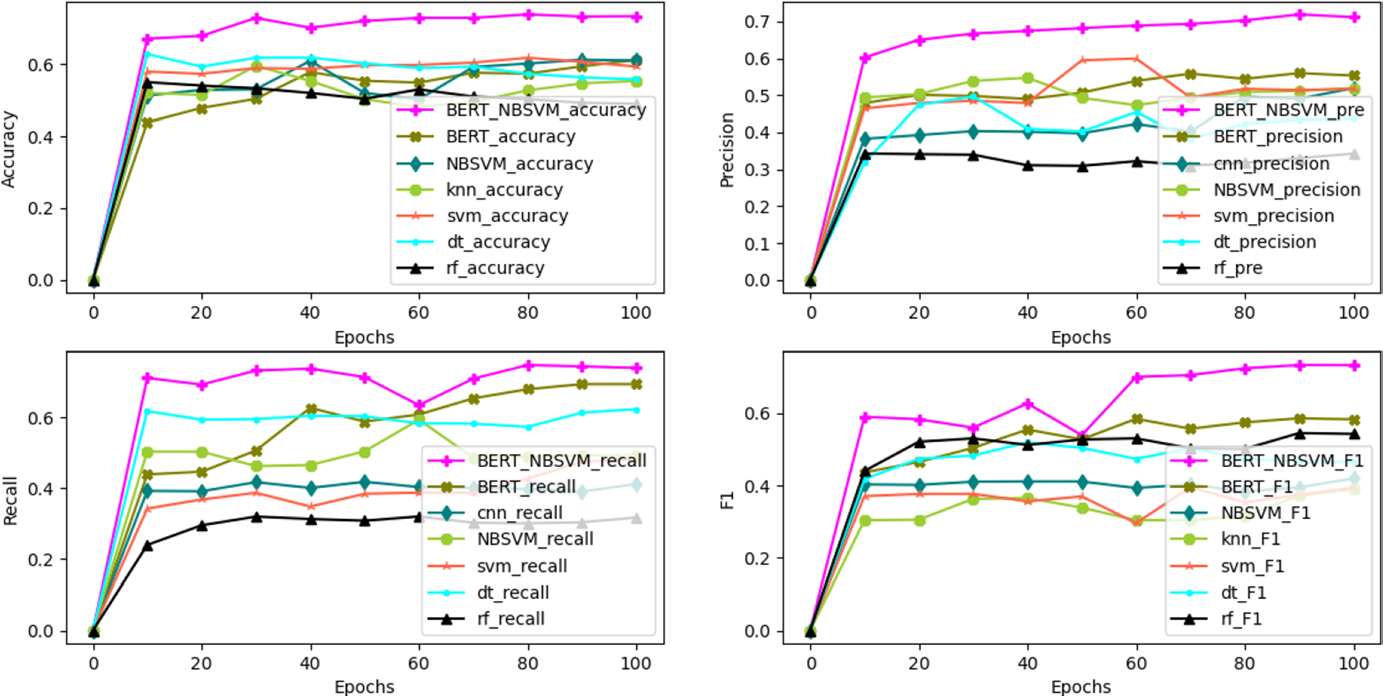
Comparison of BERT+NBSVM model with state-of-the-art for positive sentiment classification

**Fig. 9:**
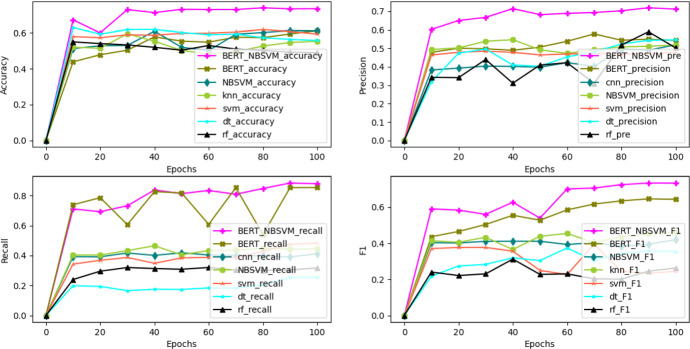
Comparison of BERT+NBSVM model with state-of-the-art for negative sentiment classification

The results of the experiments are reported in Figs. 8 and 9.

Figure 8 shows sub-graphs depicting the classification accuracy, precision, recall, and *F1* score of our BERT+NBSVM model compared to BERT, NBSVM, decision trees, KNN, random forest, and SVM for the classification of positive sentiments. The results show that our approach outperformed all other state-of-the-art models by achieving the highest accuracy.^8^

Figure 9 shows the plots of Accuracy, Precision, Recall and *F1* score for our ] model compared to BERT, NBSVM, Decision tree, KNN, random forest and SVM for the classification of negative sentiments. Again our BERT+NBSVM model exhibits best performance among all other state of the art neural network and machine learning models we implemented.

To wrap up, the qualitative factors that lead to better BERT+NBSVM performances can be summarized as follows:

- Feature engineering computation: NBSVM is able to extract relevant information from the text using feature engineering, thus BERT shows better feature engineering when equipped with NBSVM.
- Complementary strengths: NBSVM is well-suited for handling large datasets while BERT is well-suited for identifying the semantics of texts. Combining these models results in a synergy.
- Transfer learning: BERT is pre-trained on large texts and then fine-tuned on a specific task. Pre-training is a form of transfer learning, and when combined with NBSVM, it achieves good performance.

When considering classical machine learning models, the performance of SVM is higher than other baseline algorithms because SVM does not show any side-effects of hyper-parameters related to the data [47]. KNN and decision trees show similar accuracy, and they have a significant impact on sentiment classification [48]. Finally, Random Forest shows intermediate performance in both scenarios of our experimental setting because Random Forest draws observation strategies randomly and requires hyper-parameter tuning to achieve better performance [49].

## Discussing geo-spatial analysis of COVID vaccine tweets

Recently, social media data has been used for GIS-based modeling of infectious diseases [13]. The term GIS stands for Geographical Information System, which is a technology used for capturing, managing, analyzing, and presenting spatial or geographical data. It is widely used in several fields, including earth sciences, urban planning, natural resource management, and public health. GIS-based disease modeling refers to the identification of key features such as the location of disease occurrence, the intensity of the disease in a specific location, the pattern of disease spread, and the eventual spatial relationship among affected areas. In [50], the authors monitored and estimated the spread of epidemics in the real world by using Cellular Automata (CA). In [51], a review of 63 articles that used GIS-based approaches to find the distribution patterns of COVID-19 is presented. Moreover, in [52], the authors worked on informed decision-making in the COVID-19 pandemic using GIS-based approaches. Despite the introduction of new techniques, the importance of GIS-based modeling is still high [53] because we need to collect and share disease data since infections are highly dependent on spatial factors.

In this section, we describe our approach to refining the vaccine center indications taking into account the findings on the people’s sentiment thus acting as a kind of specialized recommender system, as this kind of systems are extensively studied nowadays especially when they take advantage of well established tools like GIS.

### Vaccine hesitancy due to access issues

Based on the results of the word clouds presented in Sect. 5.2, it can be noted that sentiment analysis demonstrates a low willingness to vaccinate. Therefore, the use of geospatial analysis to identify potential barriers to vaccination can be fruitful. For some individuals, vaccine hesitancy may be driven by barriers to accessing vaccines, such as lack of transportation, long wait times, or difficulty scheduling appointments. The report by the WHO Strategic Advisory Group of Experts (SAGE) on Immunization identifies confidence (lack of trust towards vaccine providers), complacency (unable to understand the importance of a vaccine for a particular disease), and convenience (access to vaccines i.e. physical availability, geographical accessibility, and the ability to understand because of issues with language or health literacy affecting uptake) as the three main factors influencing vaccine hesitancy [54]. Geospatial approaches can address the third factor i.e., convenience or access to vaccination by suggesting proper vaccination centers to people based on sentiment analysis results in a given area.

### Geo-coding and visualization of data

**Fig. 10:**
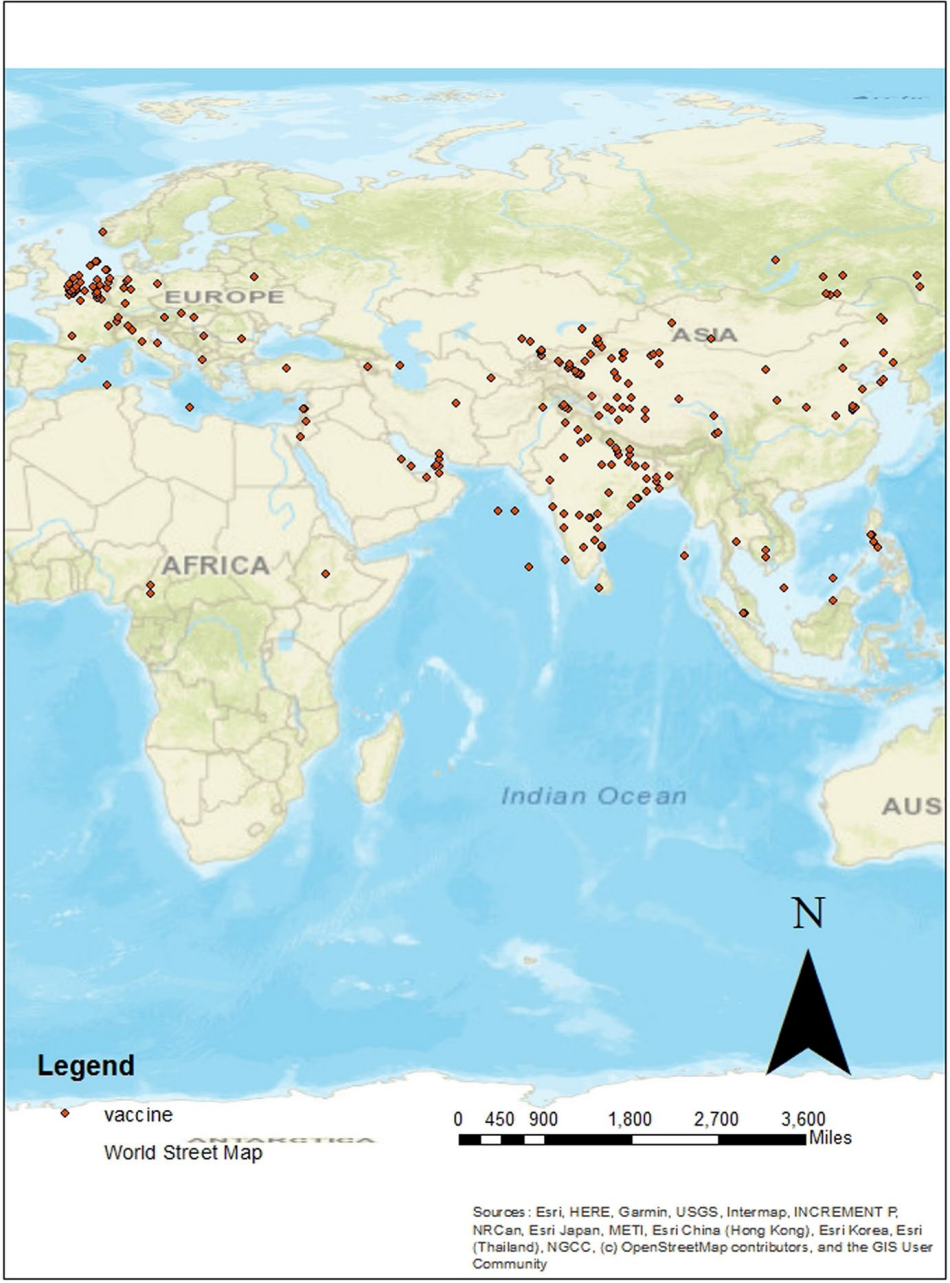
Vaccine data visualization

We partitioned our dataset into subsets and applied geocoding to each instance. Geocoding is the process of converting the address of a location into its respective geographic coordinates. We used the geoPy library in Python for geocoding, which utilizes third-party geocoders to locate geographic coordinates [55]. After this step, we visualized the geocoded vaccination data on a map surface using ArcGIS 10.5. Figure 10 shows the distribution of vaccines around the world.

### Geographical correlation

Discovering relationships among features in a dataset are crucial for identifying spatial patterns. In more detail, feature points can be spatially clustered, random, or dispersed. The null hypothesis assumes that features are entirely randomly distributed. The pattern analysis we performed returned a *p*-value and *z*-score, which are used to reject the null hypothesis. Therefore, if the null hypothesis is falsified, there exists a relationship among the features. This relationship may indicate clustering or dispersion. If a clustered relationship exists, it shows high geographical associativity among the features.

### P-value and Z-score computation

*P*-value is a measure of the probability of obtaining a result as extreme as, or more extreme than, the observed result. It shows the spatial pattern of the random process and ranges between 0 and 1. Smaller values indicate that the pattern is not random. *Z*-score measures the standard deviation, which computes how the data are distributed with respect to the mean value. For example, a *Z*-score value of 0 shows that the observed value is equal to the average value, while 2.5 means that it is 2.5 away from the average. *Z*-scores can be positive or negative. A high (positive) *z*-score indicates that the data point is far above the mean, while a low (negative) *z*-score means that the data point is far below the mean. A confidence range is assigned to the range of *P*-values and *Z*-scores as shown in Table 3 [56].

**Table 3:** Confidence level associated with *P*-values and *Z*-scores

*P*-value	*Z*-score	Confidence level (%)
< 0.10	< − 1.65 or > +1.65	90
< 0.05	< − 1.96 or > +1.96	95
< 0.01	< − 2.58 or > +2.58	99

The *P*-values and *Z*-scores are computed together to test the null hypothesis. If the *Z*-score is very high or very low combined with a small *P*-value, the null hypothesis is rejected [56].

### Average nearest neighbor

We leveraged the ANN (Average Nearest Neighbor) tool of ArcGIS to identify the spatial relationship among the points in the vaccine dataset. The ANN is defined as the ratio of the observed average distance (DO) to the expected average distance (DE), and can be computed using Eq. 7.

7 $$\begin{aligned} {\rm ANN} = {\rm DO}/{\rm DE} \end{aligned}$$

**Fig. 11:**
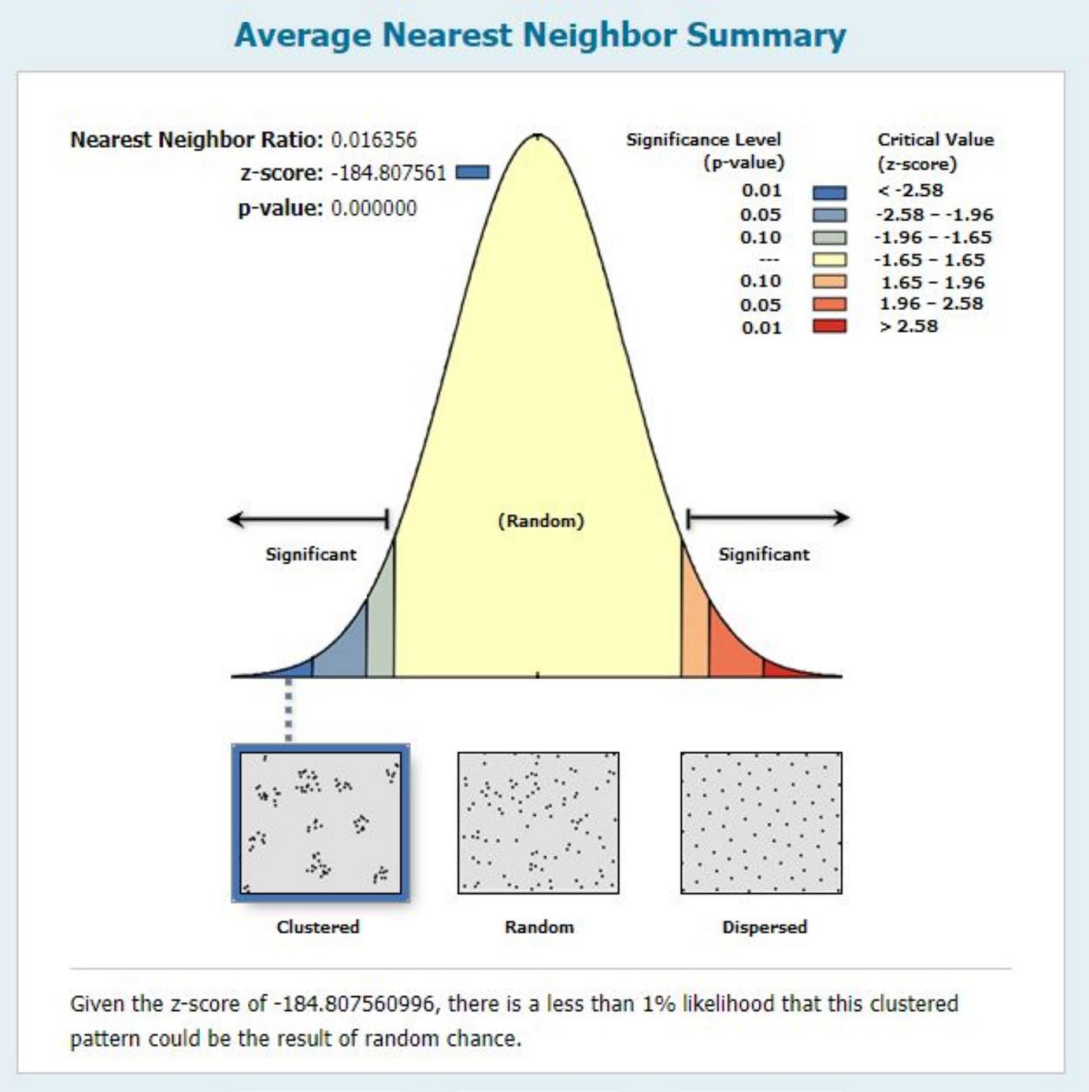
Spatial correlation among the data features

If the value of ANN is less than 1, it indicates that the dataset is clustered [56]. In our case, we obtained a value that is less than 1, confirming that our data are clustered, as shown in Fig. 11.

Furthermore, we can observe in Fig. 11 that the *Z*-score obtained for our dataset is − 184.807561, while the *p*-value is 0.000000. Upon examining the values of the *z*-score and *p*-values in Table 3, we can see that the *p*-value puts us in a 99% confidence level, while the *Z*-score puts us in a 95

### Buffering

**Fig. 12:**
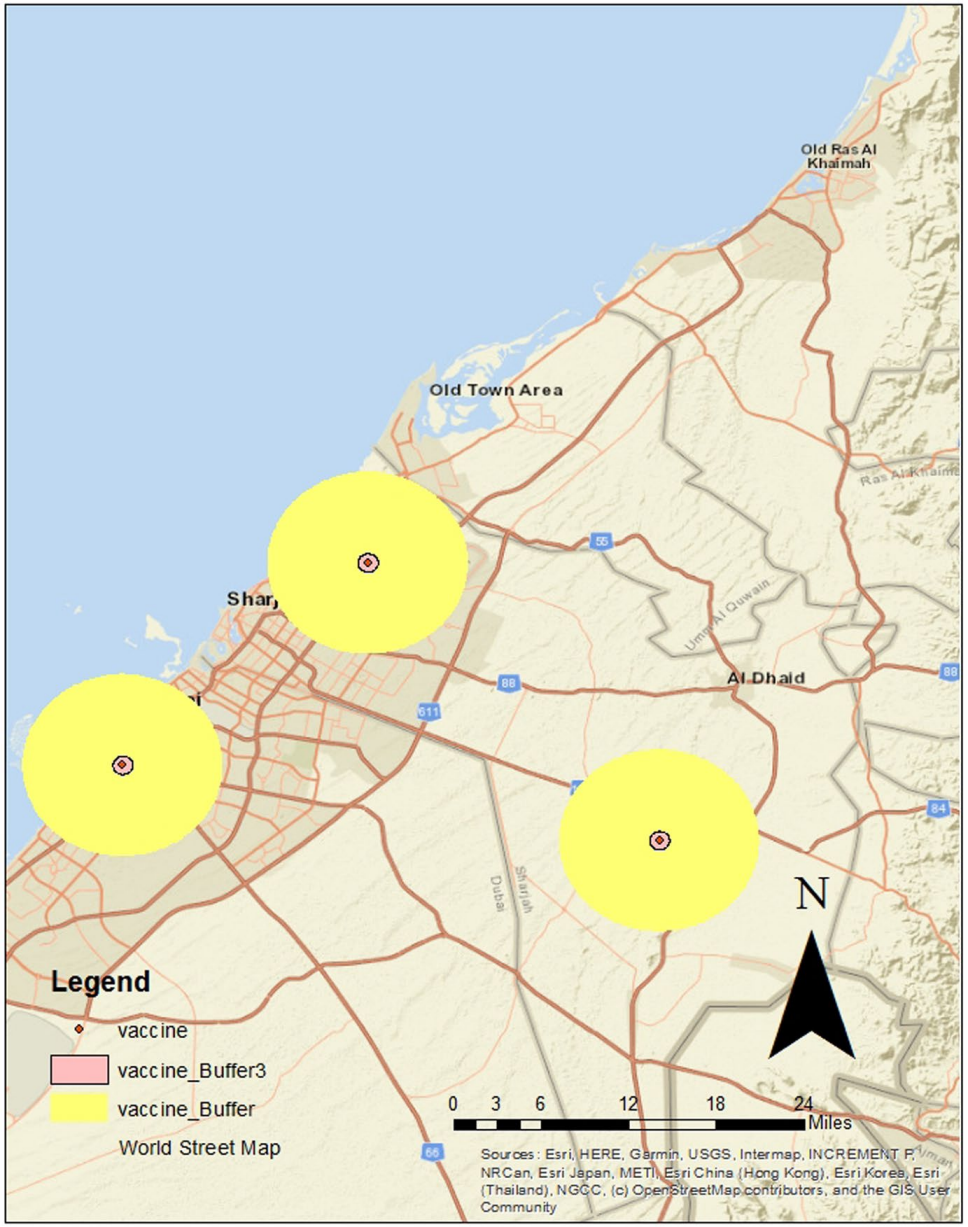
Results of buffering on vaccination dataset

We discussed in previous sections that a possible obstacle to getting vaccinated may be mobility, as defined in Sect. 6. Geo-spatial techniques, like buffering, can be efficiently used to address allocation problems [57, 58]. We performed buffering using ArcGIS to find the suitability of vaccination centers with respect to people’s locations. The buffering analysis of our data is reported in Fig. 12, which shows the willingness of people to move for vaccination. We assumed that vaccination centers must be within 10 km of the user’s address. Indeed, several scenarios were run with different distance values, and 10 km resulted in the most favored distance to encourage people to get vaccinated

Figure 12 shows the results we obtained by running buffering over the vaccination dataset. We can observe two types of buffers in the diagram. The pink buffer visualizes the nearest possible vaccination centers, while the yellow buffer shows the results on an international scale.

## Conclusion

Social media-based sentiment analysis of people’s feelings about vaccines is a useful and cost-effective way to design policies for vaccination campaigns. Moreover, the use of geo-spatial approaches, along with Twitter analysis, can help to find the most likely reasons behind vaccine hesitancy. As a matter of fact, vaccines were developed to control the spread of COVID-19 worldwide, but vaccine hesitancy seems to be an even bigger challenge than COVID-19. In this paper, we identify people’s reactions during the vaccination phase. We used Twitter data and found out the polarity of the tweets. We categorized them into seven categories, considering tweet polarity values. We proposed a BERT+NBSVM classification model for the classification of positive and negative sentiments. Our work also focuses on the usage of geo-spatial approaches to identify the geo-spatial patterns in the vaccination data. We performed buffering to suggest proper vaccination centers based on sentiment analysis. Thus, policymakers can benefit from our methods by analyzing people’s concerns and understanding their mindset to improve proper planning to inform people about vaccines, identify misinformation or rumors spreading across the country, and launch ad-hoc campaigns suited to avoid confusion on this important topic.

### Limitations and future work

COVID-19 is a global pandemic and sentiments are being expressed about it in different countries and languages. Due to the lack of reliable multilingual resources, we were only able to perform sentiment analysis on English language tweets. Moreover, proper analysis of the overall context of the tweet could be a limitation when considering different cultures as the way they express positive feelings may be very different compared to the English language. Thus, as a future research direction, we aim to explore the effectiveness of the BERT+NBSVM model for sentiment analysis using different languages. Furthermore, while sentiment analysis typically categorizes text as positive, negative, or neutral, there could exist a more refined classification of people’s feelings about COVID-19, considering that tweets often incorporate images. Fine-grained sentiment analysis could help capture this set of nuances by identifying specific emotions or attitudes expressed in text and pictures. In this respect, we will work in the near future on the application of emotion-based analysis to Tweets related to COVID-19.

Furthermore, our BERT+NBSVM model has shown compelling performance on sentiment analysis tasks related to COVID-19, but it may not generalize well to other domains. Future research will explore how to adapt the model to other domains while still maintaining the effectiveness proven for COVID-related sentiment analysis. Indeed, transfer learning is a powerful technique that can improve the performance of sentiment analysis models by leveraging pre-trained models on large datasets. Future research will explore how to use transfer learning to improve their performance on health-related sentiment analysis tasks. Finally, sentiment analysis can be valuable in real-time monitoring of public sentiment about COVID-19. Future research could explore the optimization of BERT+NBSVM model for real-time sentiment analysis in social media.

## Data Availability

The datasets generated during and/or analysed during the current study are available from the corresponding author on reasonable request.

## Footnotes

1 https://www.kaggle.com/datasets/gpreda/all-covid19-vaccines-tweets.

2 https://hbase.apache.org.

3 https://spark.apache.org/.

4 https://www.mongodb.com/.

5 http://storm.apache.org/.

6 Streaming data was not accessible for our experimental assessment.

7 This assumption may be controversial as the context in which these words are used plays a crucial role. Indeed, in our dataset, we leveraged some statistical analyses that suggest that, in our case, these terms may indicate a positive attitude towards getting vaccinated.

8 We would like to emphasize that we performed our comparison under the same race conditions for all approaches, using the same dataset. Therefore, our superior performance can be attributed to the fact that our hybrid model refines the results obtained by classical algorithms, improving intermediate results computed by BERT and SVM.
